# Comparative Analysis of Support Vector Machine Variants for Human Activity Recognition Using Wearable Sensor Data

**DOI:** 10.3390/s26175551

**Published:** 2026-09-01

**Authors:** Minh Long Hoang

**Affiliations:** Department of Engineering and Architecture, Faculty of Electronic Engineering, University of Parma, 43124 Parma, Italy; minhlong.hoang@unipr.it

**Keywords:** Human Activity Recognition, Support Vector Machine, Multiple Kernel Learning, kernel methods, wearable sensors, hyperparameter optimization

## Abstract

Human Activity Recognition (HAR) using wearable sensor data is widely applied in healthcare, smart environments, and mobile systems. Support Vector Machines (SVMs) are commonly used for HAR due to their strong generalization ability. However, traditional approaches often rely on single kernels and may not fully capture complex motion patterns. This research presents a comparative analysis of seven SVM-based methods, including Multiclass SVMs, Radial Basis Function (RBF) SVMs, Kernel Engineering SVMs, Multiple Kernel Learning (MKL) SVMs, Class-Weighted SVMs, Least Squares SVM approximation, and Online Incremental SVMs. A unified experimental framework with consistent preprocessing and hyperparameter tuning using Grid Search with cross-validation is employed to ensure fair evaluation. Results show that kernel-based methods outperform linear and approximate models. The MKL SVM achieves the highest accuracy, slightly surpassing the RBF baseline, by combining multiple kernels to capture diverse data characteristics. Kernel Engineering SVM also improves performance, while Fuzzy and LS-SVM provide competitive results with enhanced robustness. In contrast, Multiclass and Online SVM exhibit lower accuracy. Thes results demonstrate that improving feature representation through advanced kernel design is key to enhancing HAR performance.

## 1. Introduction

Human Activity Recognition (HAR) [1,2,3] has emerged as a critical research area in machine learning and ubiquitous computing, driven by its wide range of applications in healthcare monitoring, smart environments, wearable devices, and human–computer interaction. HAR systems aim to automatically identify physical activities such as walking, sitting, stair climbing/going down, using data collected from sensors including accelerometers and gyroscopes. With the rapid growth of Internet of Things (IoT) technologies and mobile sensing platforms, accurate and efficient HAR has become increasingly important for real-time intelligent systems [4,5]. Among various machine learning techniques, Support Vector Machines (SVMs) [6,7] have been widely adopted for HAR due to their strong theoretical foundation, robustness to high-dimensional data, and good generalization performance. Traditional SVM models, particularly those using linear and Radial Basis Function (RBF) kernels [8], have demonstrated competitive results in smartphone-based HAR tasks [9]. However, real-world HAR data present several challenges, including nonlinear motion patterns, sensor noise, class overlap, and heterogeneous feature distributions from different sensing modalities [10]. To address these limitations, several advanced SVM variants have been proposed. Multiclass SVM approaches extend the original binary SVM formulation to handle multiple activity classes using strategies such as One-vs-One (OvO) [11], One-vs-Rest (OvR) [12], and direct multiclass optimization methods like the Crammer–Singer formulation [13]. Kernel-based approaches further enhance SVM performance by transforming input data into higher-dimensional feature spaces, enabling the modeling of complex nonlinear relationships [14].

More sophisticated methods such as Multiple Kernel Learning (MKL) combine multiple kernel functions to capture diverse characteristics of the data [15]. By integrating linear, polynomial, and RBF kernels, MKL provides a richer and more flexible representation, which has shown improved performance in various classification tasks including activity recognition. Similarly, Kernel Engineering techniques design customized kernels tailored to specific sensor modalities, allowing better exploitation of heterogeneous data sources. Class-Weighted SVM [16] introduces sample weighting mechanisms to reduce the impact of noisy or uncertain data points, which are common in wearable sensor signals [17,18]. Least Squares SVM (LS-SVM) [19] reformulates the standard SVM optimization problem into a set of linear equations, significantly reducing computational complexity while maintaining competitive performance. Additionally, online or incremental SVM methods [20] enable continuous model updates for streaming data, making them suitable for real-time HAR applications. Despite the availability of these advanced SVM variants, most existing studies focus on individual methods or limited comparisons, typically involving only standard SVM models or basic multiclass strategies. Anguita et al. [9] proposed a multiclass hardware-friendly SVM for smartphone-based HAR. Although the method achieved promising recognition performance, the study mainly focused on reducing computational cost for mobile devices rather than investigating advanced Kernel Learning strategies. Furthermore, the work relied on conventional multiclass decomposition techniques and did not explore adaptive kernel combinations or robustness-oriented SVM variants. Falcari et al. [21] evaluated One-vs-One (OvO) and One-vs-All (OvA) Multiclass SVM strategies using linear, polynomial, and RBF kernels. Their work demonstrated that kernel selection affects classification performance; however, the study remained limited to conventional decomposition strategies and individual kernels without investigating kernel fusion or advanced SVM architecture. Moreover, previous surveys on SVM classification methods [22] highlighted that kernel selection strongly influences classification performance and emphasized the importance of Multiple Kernel Learning approaches. Nevertheless, many existing HAR studies still employ only a single kernel function, which may not adequately represent heterogeneous sensor characteristics.

Compared with previous studies, the present work provides several important contributions. First, instead of focusing on a single SVM configuration, this study performs a unified comparison of seven advanced SVM variants under identical experimental conditions based on Inertial Measurement Unit sensors [23,24,25,26], specifically the accelerometer and gyroscope [27,28,29,30]. Second, the work investigates both representation-oriented approaches (Kernel Engineering and MKL) and robustness-oriented approaches (Class-Weighted SVM, LS-SVM, and Online SVM). Third, unlike many previous HAR studies that employ only individual kernels, this work demonstrates the effectiveness of combining multiple kernels for wearable sensor-based activity recognition. Finally, systematic hyperparameter optimization using Grid Search and cross-validation ensures a fair and reproducible comparison across all models. Comprehensive comparative studies that evaluate multiple advanced SVM approaches under a unified HAR framework are still limited. Therefore, this work addresses this gap by providing a systematic and comprehensive comparison of multiple advanced SVM approaches for HAR. The main contributions are as follows:

- A unified experimental framework is developed to evaluate seven SVM-based methods under identical preprocessing, training, and evaluation conditions.
- In contrast to existing studies that mainly focus on conventional SVM models, this work compares the following: Multiclass SVM, RBF SVM (baseline), Kernel Engineering SVM, MKL SVM, Class-Weighted SVM, Least Squares SVM (approximation), and Online/Incremental SVM.
- A custom Kernel Engineering strategy is proposed, combining nonlinear kernels for accelerometer data with linear kernels for gyroscope data to better capture heterogeneous sensor characteristics.
- A Multiple Kernel Learning-style framework is implemented to integrate multiple kernels, enabling richer feature representation and improved classification performance.
- Hyperparameter optimization is systematically performed using GridSearchCV and structured search strategies, ensuring a fair and unbiased comparison.
- The study provides detailed performance evaluation using validation accuracy, test accuracy, classification reports, and confusion matrices.
- The results demonstrate that enhancing feature representation through kernel design is more effective than simply increasing model complexity, providing valuable insights for future HAR system design.

The remainder of the paper is organized as follows: The next chapter presents a description of the wearable setup and SVM methods; the chapter that follows details the result analysis; and finally, the discussion and conclusion are presented.

## 2. Materials and Methods

**Figure 1:**
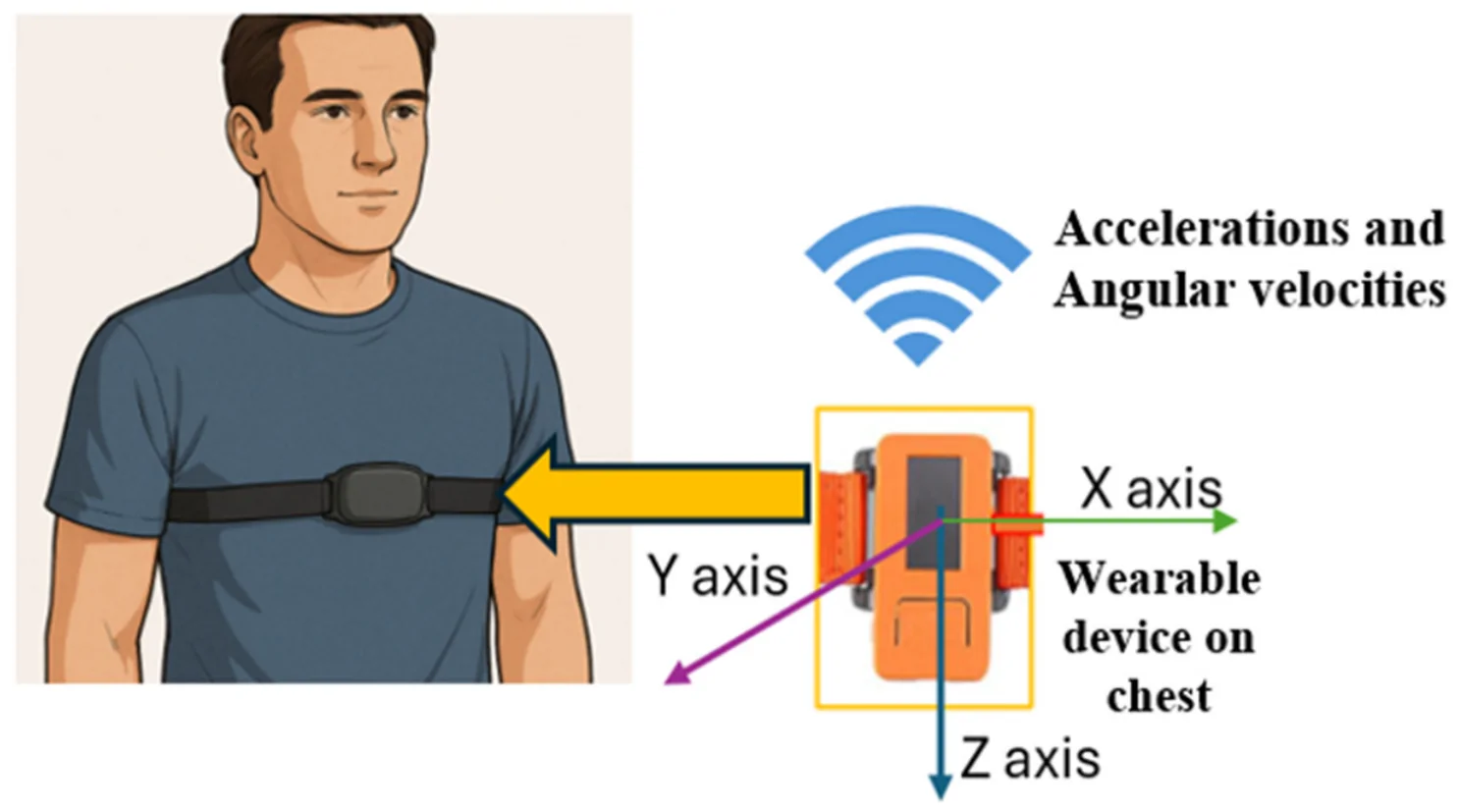
Wearable device setup.

As demonstrated in Figure 1, a wearable M5StickC PLUS2 [31] was equipped on the user’s chest to acquire the triaxial acceleration (Xacc, Yacc, Zacc) and triaxial angular velocities (Xgy, Ygy, Zgy) corresponding to 4 activities: Climb Stairs, Go Down Stairs, Sit Down, and Walk. The sensing device was securely strapped to the participant’s chest using an adjustable elastic band to minimize movement relative to the body during activity execution. The device was positioned at the center of the chest with a consistent orientation across all participants and trials. The 6 degrees-of-freedom (DOF) Inertial Measurement Unit (IMU) is embedded inside this device, with 16-bit Analog-to-Digital Converters (ADCs) to sample each axis. The full-scale range of the accelerometer and gyroscope are set at ±2 g and ±500 °/s respectively. The data acquisition frequency is 10 Hz. A sampling frequency of 10 Hz was adopted because the dominant motion components associated with the concerned activities occur within a low-frequency range and can be adequately captured according to the Nyquist criterion. In addition, the lower sampling rate reduces the computational and energy requirements, making it suitable for wearable healthcare-monitoring applications. Meanwhile, higher sampling frequencies increase energy consumption and require more storage space for data. For these activities, the 10 Hz sampling rate preserves the principal temporal and inertial characteristics required for classification while avoiding unnecessary oversampling and data redundancy. At this sampling frequency, the data can be fed directly to the ML model. Each classification sample consisted of one six-dimensional IMU observation received every 0.1 s. The calibration of the IMU device was carried out, and the bias and offset were removed. For the accelerometer, the calibration was calibrated based on the body frame and Earth’s gravity. For the gyroscope, the drift was calculated based on the additional angular velocities observed when the sensor remained stationary, which were then removed.

Three participants with ages ranging from 30 to 40 years old provided about 10,700 samples. Each person carried out approximately 26 s of Climb Stairs, 47 s of Go Down Stairs, 109 s of Sit Down, and 174 s of Walk. Dataset samples were split as follows: 70% for training, 10% for validation, and 20% for test. The same data partitions were consistently used for all SVM-based methods. This partitioning strategy was selected in accordance with the primary objective of this study, which is to provide a controlled comparison of different SVM formulations and Kernel Learning strategies for HAR. Using identical training, validation, and test samples for all models ensures that the observed differences in classification performance are primarily associated with the characteristics of the investigated algorithms rather than differences in the data provided to each model. The validation data were used during model development, whereas the independent test subset was used for final performance evaluation. Therefore, the present evaluation focuses on the comparative performance of the SVM methods in a mixed-participant setting. Participant-independent evaluation, in which activities from an unseen participant are exclusively reserved for testing, represents a complementary protocol for investigating cross-subject generalization and will be considered in future work.

This work investigates and compares seven SVM-based approaches for HAR. All methods are evaluated under a unified experimental framework, including identical preprocessing, data splitting, and evaluation metrics, to ensure a fair comparison.

Device safety: The prototype was implemented using an M5StickC PLUS2 development board (M5Stack Technology Co., Ltd., Shenzhen, China), which is a commercially available electronic device intended for consumer and educational applications. The M5StickC PLUS2 is a commercially available development platform certified for use in multiple regulatory jurisdictions. According to the manufacturer, the device carries CE (European Conformity), FCC (Federal Communications Commission), and Ministry of Internal Affairs and Communications (MIC) certifications, demonstrating compliance with applicable requirements for radio-frequency emissions, electromagnetic compatibility (EMC), and wireless communication equipment. In addition, the integrated rechargeable battery complies with IEC 62133 [32], an international safety standard for portable rechargeable batteries. These certifications guarantee that the hardware has undergone regulatory conformity assessment for electrical, electromagnetic, wireless, and battery-related safety requirements.

- M5Stack Technology Co., Ltd. M5Stack Products Certification. Available online: https://docs.m5stack.com/en/learn/certification/certification (accessed on 20 February 2026).
- Federal Communications Commission (FCC). FCC ID: 2AN3WSTICKCPLUSV11—M5StickC PLUS2. Available online: https://fccid.io/2AN3WSTICKCPLUSV11 (accessed on 20 February 2026).

### 2.1. Multiclass SVM

Traditional SVM is inherently a binary classifier. To address multiclass HAR problems, this study employs a direct multiclass formulation based on the Crammer–Singer approach [7]. Unlike decomposition strategies such as One-vs-One (OvO) and One-vs-Rest (OvR), this method optimizes a single objective function across all classes simultaneously.

Given an input feature vector x, the decision function is defined as

(1) $$f(x)=arg\;\underset{k}{max}(w_{k}^{T}x+b_{k})$$

where $w_{k}$ and $b_{k}$ represent the weight vector and bias for class k. This formulation ensures global optimization across all classes and avoids inconsistencies associated with combining multiple binary classifiers.

### 2.2. RBF SVM (Baseline Model)

The Radial Basis Function (RBF) kernel SVM is used as the primary baseline due to its strong capability to model nonlinear relationships. The RBF kernel maps input data into a high-dimensional feature space:

(2) $$K(x_{i},x_{j})=exp(-\gamma ∥x_{i}-x_{j}{∥}^{2})$$

where γ controls the kernel width. The RBF SVM is particularly effective for HAR tasks because human motion patterns are inherently nonlinear.

### 2.3. Kernel Engineering SVM

To better exploit the heterogeneous nature of sensor data, a custom Kernel Engineering strategy is proposed. The feature space is divided into the following:

- Accelerometer features (nonlinear behavior);
- Gyroscope features (approximately linear behavior).

The selection of sensor-specific kernels was introduced as a Kernel Engineering strategy to investigate complementary representations of the two inertial sensing modalities. The accelerometer features were modeled using an RBF kernel to provide a flexible nonlinear representation of translational body-motion patterns, whereas the gyroscope features were represented using a linear kernel as a simpler representation of rotational-rate information. This configuration represents an experimentally evaluated sensor-specific kernel design. The two kernel components were subsequently combined using weighted kernel fusion. The combined kernel is defined as

(3) $$K=\alpha K_{\mathrm{RBF}}^{\mathrm{acc}}+(1-\alpha )K_{\mathrm{linear}}^{\mathrm{gyro}}$$

where α∈[0,1] controls the contribution of each sensor modality. This approach allows the model to capture both nonlinear motion dynamics and linear rotational patterns.

### 2.4. Multiple Kernel Learning (MKL) SVM

Multiple Kernel Learning extends the idea of Kernel Engineering by combining multiple kernel functions into a single unified representation:

(4) $$K=w_{1}K_{\mathrm{linear}}+w_{2}K_{\mathrm{RBF}}+w_{3}K_{\mathrm{poly}}$$

subject to

(5) $$w_{1}+w_{2}+w_{3}=1;\;w_{i}\geq 0$$

where (w_1_), (w_2_), and (w_3_) represent the contributions of the linear, RBF, and polynomial kernels, respectively.

The combination enables the classifier to simultaneously exploit linear relationships captured by the linear kernel, local nonlinear patterns represented by the RBF kernel, and higher-order feature interactions modeled by the polynomial kernel. In the present implementation, the kernel weights were optimized through a structured validation-based search rather than jointly learned with the SVM classifier. Specifically, predefined weight combinations for ($w_{1}$, $w_{2}$, $w_{3}$) were evaluated together with the SVM hyperparameters (C), γ, and polynomial degree. For each candidate configuration, a composite kernel matrix was constructed and used to train an SVM with a precomputed kernel. The configuration yielding the highest validation accuracy was selected as optimal.

This procedure differs from classical MKL formulations, in which kernel weights are treated as continuous optimization variables and are learned jointly or iteratively with the classifier parameters. Therefore, the method implemented in this study can more precisely be described as a weighted multiple-kernel SVM with structured kernel-weight optimization. It retains the principal advantage of combining complementary kernel representations while providing a transparent and computationally manageable procedure for selecting their relative contributions.

### 2.5. Class-Weighted SVM

Class-Weighted SVM introduces sample-dependent weights to reduce the influence of noisy or uncertain data points. Each training sample is assigned a membership value $\mu_{i}\in [0,1]$, modifying the standard SVM objective:

(6) $$\min\frac{1}{2}∥w{∥}^{2}+C\sum_{i}\mu_{i}\xi_{i}$$

subject to

(7) $$y_{i}\left(w^{T}\phi \left(x_{i}\right)+b\right)\geq 1-\xi_{i}$$

where *w* is the SVM weight vector, *C* is the regularization parameter, $\xi_{i}$ represents the slack variable ${(\xi }_{i}>0)$, $\mu_{i}$ is the class-dependent weight assigned to sample *i*, $\phi \left(x_{i}\right)$ is feature-space transformation, and *b* is bias term.

In this work, the weights are determined using the balanced class-weighting scheme as follows:

(8) $$\mu_{i}=\;\frac{N}{KN_{K}}$$

where N is the total number of training samples, K is the number of classes, and $N_{K}$ is the number of training samples belonging to the class of sample. Based on the results, samples from less represented classes receive higher weights, whereas samples from more represented classes receive lower weights. This process increases the penalty associated with misclassifying samples from minority classes and reduces the influence of class imbalance on the learned decision boundary.

### 2.6. Least Squares SVM (LS-SVM) Approximation

Least Squares SVM reformulates the traditional SVM optimization problem into a Least Squares problem, replacing inequality constraints with equality constraints. The objective becomes

(9) $$\min\frac{1}{2}∥w{∥}^{2}+\frac{C}{2}\sum_{i}e_{i}^{2}$$

where $e_{i}$ is the error term.

In this study, LS-SVM is approximated using the following:

- RBF kernel feature mapping (via Nystroem approximation);
- Ridge regression classifier.

This approach reduces computational complexity while preserving the nonlinear modeling capability, making it suitable for large-scale HAR datasets.

### 2.7. Online/Incremental SVM

The Online SVM approach is implemented using stochastic gradient descent (SGD) with hinge loss:

(10) $$\min\frac{1}{2}∥w{∥}^{2}+C\sum_{i}\max(0,1-y_{i}(w^{T}x_{i}))$$

Unlike batch SVM methods, this model updates its parameters incrementally as new data arrives, enabling the following:

- Continuous learning;
- Adaptation to changing user behavior;
- Efficient handling of streaming sensor data.

Although it may sacrifice some accuracy compared to batch methods, it offers significant advantages in scalability.

### 2.8. Hypermeter Optimization

Hyperparameter optimization for all SVM-based models was performed using a systematic Grid Search strategy. Grid Search is an exhaustive search technique that evaluates model performance across a predefined set of hyperparameter combinations to identify the optimal configuration. In this study, the implementation was carried out using the GridSearchCV framework, which integrates cross-validation into the parameter search process. For each model, a discrete search space was defined for the key hyperparameters. In particular, the regularization parameter C, which controls the trade-off between margin maximization and classification error, varied over the set $\left\{0.01,0.1,1,10\right\}$ For kernel-based models, the kernel coefficient γ, which determines the influence of individual training samples in the feature space, was explored over the range $\left\{0.001,0.01,0.1,1\right\}$.

The evaluation of each hyperparameter combination was conducted using 5-fold cross-validation on the training set. In this process, the training data were partitioned into five subsets, where four subsets were used for training and one subset for validation. This procedure was repeated five times, ensuring that each subset was used once for validation. The average classification accuracy across the five folds was used as the performance metric for selecting the optimal hyperparameters. For models involving standard kernels like RBF SVM, Multiclass SVM, Class-Weighted SVM, Least Squares SVM approximation, and Online Incremental SVM, GridSearchCV [33] was directly applied within a pipeline framework, allowing simultaneous optimization of preprocessing (feature scaling) and model parameters. In contrast, for models using precomputed kernels, such as Kernel Engineering SVM and MKL SVM, a manual Grid Search strategy was employed. This is because GridSearchCV does not natively support dynamic kernel construction during training. In these cases, kernel matrices were explicitly computed for each combination of parameters such as kernel weights, γ, and polynomial degree, and model performance was evaluated on the validation set to identify the best configuration. The final selected hyperparameters correspond to the combination that maximized cross-validation accuracy. These optimal models were then evaluated on a held-out test set to assess their generalization performance.

Practically, the use of Grid Search with cross-validation ensures a fair and robust comparison among all SVM variants by systematically exploring the parameter space and minimizing the risk of overfitting to a specific configuration. The best hypermeters of each model were reported in Table 1.

## 3. Results

### 3.1. Comparison of Validation and Test Accuracy

**Figure 2:**
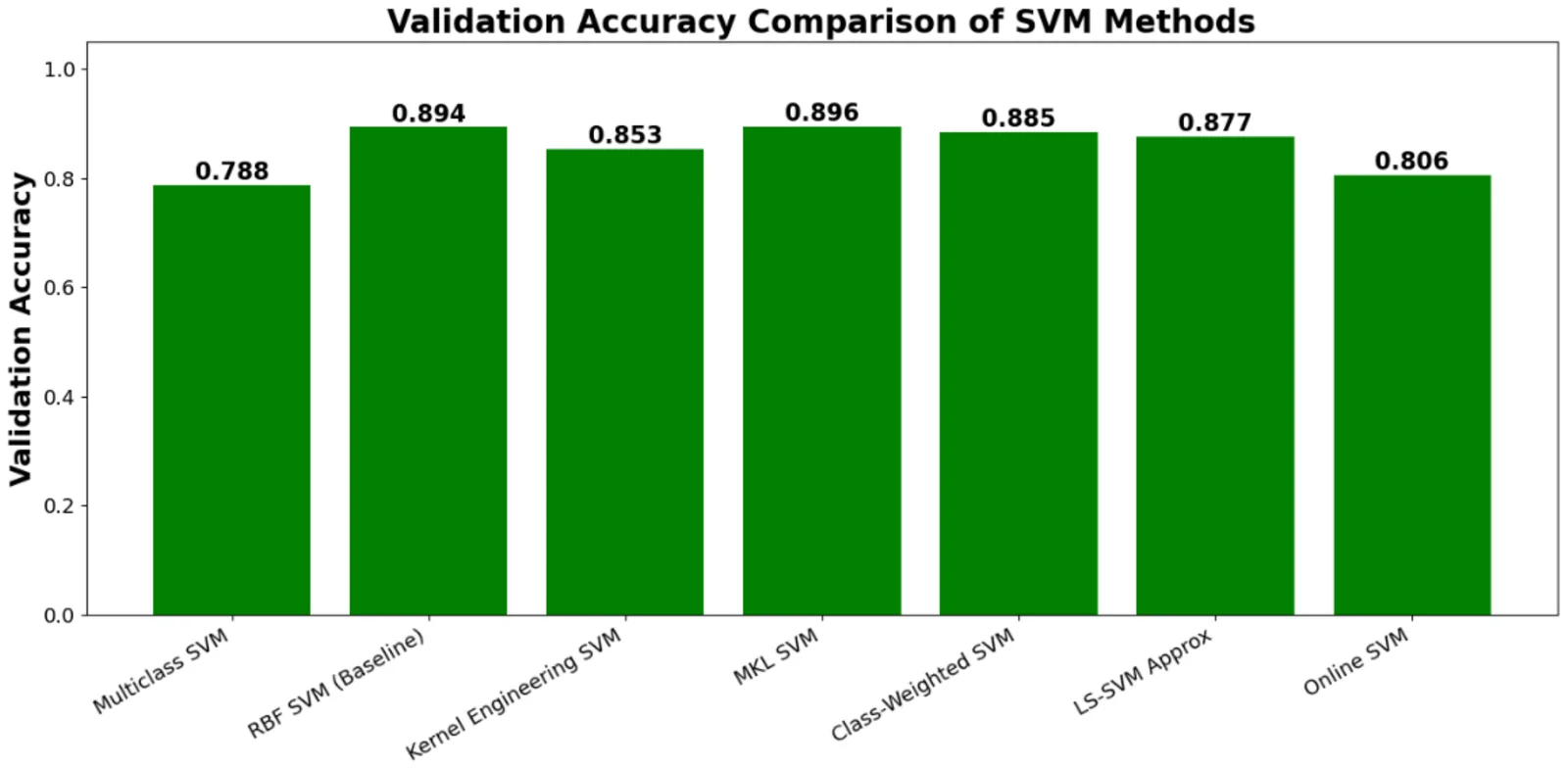
Validation accuracy comparison.

**Figure 3:**
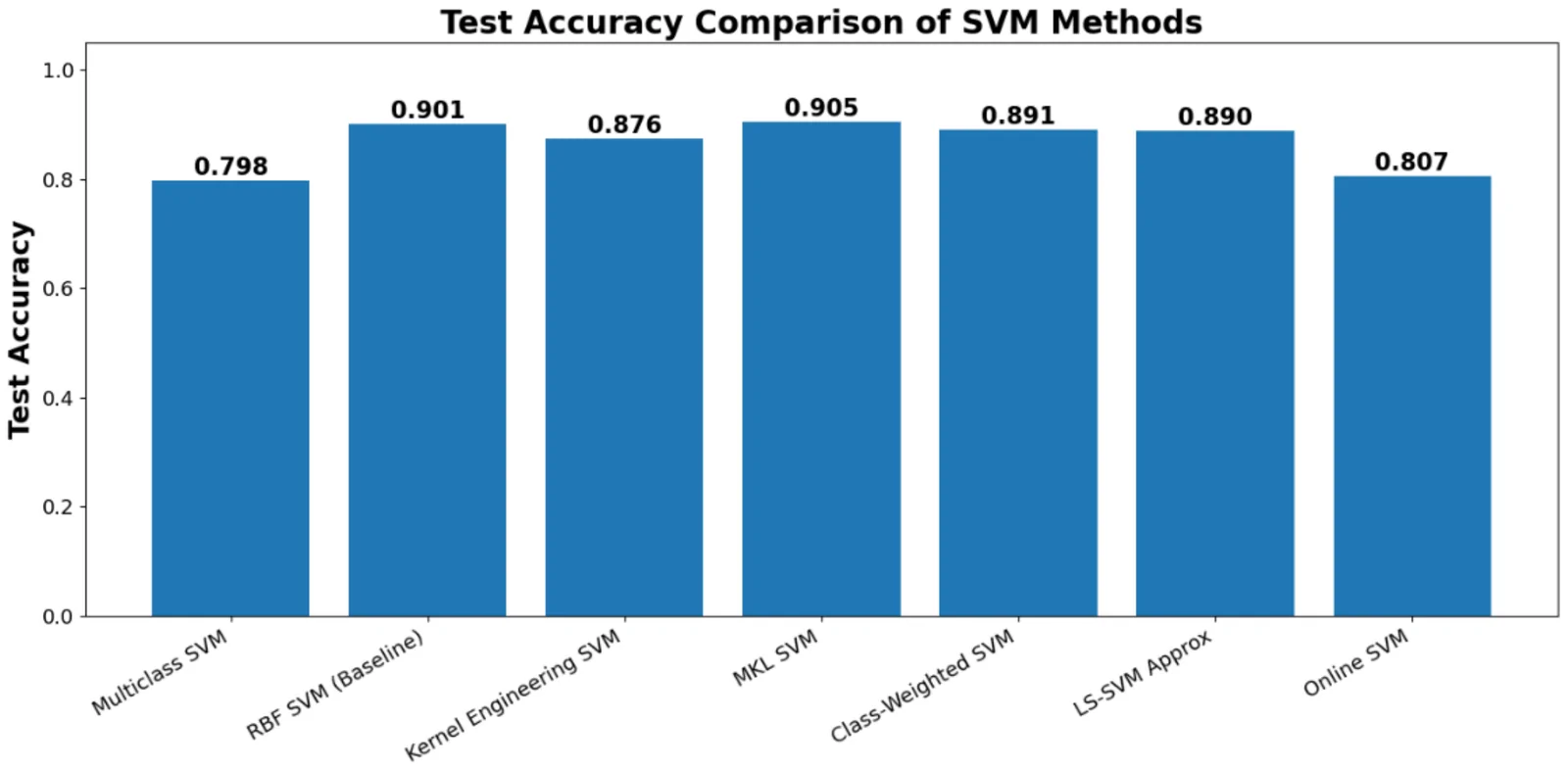
Test accuracy comparison.

As demonstrated in Table 2 and Figure 2 and Figure 3, the experimental results demonstrate clear differences in the effectiveness of the evaluated SVM-based approaches for HAR. Among the seven methods, the MKL SVM achieved the best overall performance, with a validation accuracy of 89.62% and a test accuracy of 90.55%, slightly outperforming the RBF SVM baseline, which achieved 90.08% test accuracy. This finding indicates that while the RBF kernel is already highly effective in modeling nonlinear patterns in HAR data, the combination of multiple kernels can provide additional performance gains. Although the absolute difference between the two best-performing methods is relatively small, a modest improvement in classification accuracy should not necessarily be regarded as negligible in the HAR field. Once classification performance approaches a relatively high level, further improvements generally become increasingly difficult to obtain. Moreover, HAR systems are relevant to applications such as health monitoring, assisted living, rehabilitation, and activity assessment, where reductions in activity misclassification can be practically valuable when systems operate repeatedly or over large numbers of observations. From this perspective, even a modest improvement may represent a useful algorithmic gain, particularly when it is obtained consistently without changing the dataset or evaluation conditions.

The strong performance of the RBF SVM confirms that HAR data are inherently nonlinear and cannot be effectively separated using linear decision boundaries. This is further supported by the relatively low performance of Multiclass SVM, which achieved only 79.83% test accuracy. As a linear model, it lacks the capacity to capture complex motion patterns present in accelerometer and gyroscope signals. Similarly, the Online Incremental SVM achieved 80.67% test accuracy, reflecting the trade-off between computational efficiency and predictive performance. The use of stochastic gradient descent and incremental updates limits its ability to learn highly discriminative decision boundaries compared to batch-trained kernel methods.

The MKL SVM outperformed all other methods due to its ability to integrate multiple kernel functions, including linear, RBF, and polynomial kernels, into a unified framework. This combination enables the model to simultaneously capture global linear trends, localized nonlinear relationships, and higher-order feature interactions. The optimal configuration, with C = 10 and γ = 1, and polynomial degree equal to 2, suggests that both nonlinear and interaction effects contribute significantly to classification performance. These results highlight the importance of enriching feature representation rather than relying on a single kernel function.

The Kernel Engineering SVM achieved a test accuracy of 87.55%, outperforming linear models but falling short of both the RBF and MKL approaches. This outcome indicates that while incorporating domain knowledge through sensor-specific kernel design improves performance, a fixed kernel structure is less flexible than the adaptive combination used in MKL. In contrast, the Class-Weighted SVM and Least Squares SVM approximation achieved comparable performances of 89.14% and 89.00%, respectively. These methods enhance robustness to noise and computational efficiency but do not fundamentally improve feature representation, which explains their slightly lower performance compared to the RBF and MKL models.

It is also noteworthy that the difference between validation and test accuracy is minimal across all models, suggesting good generalization and the absence of significant overfitting. This reflects the effectiveness of the hyperparameter tuning strategy and the stability of the models across unseen data.

### 3.2. Confusion Matrix and Recall Analysis

**Figure 4:**
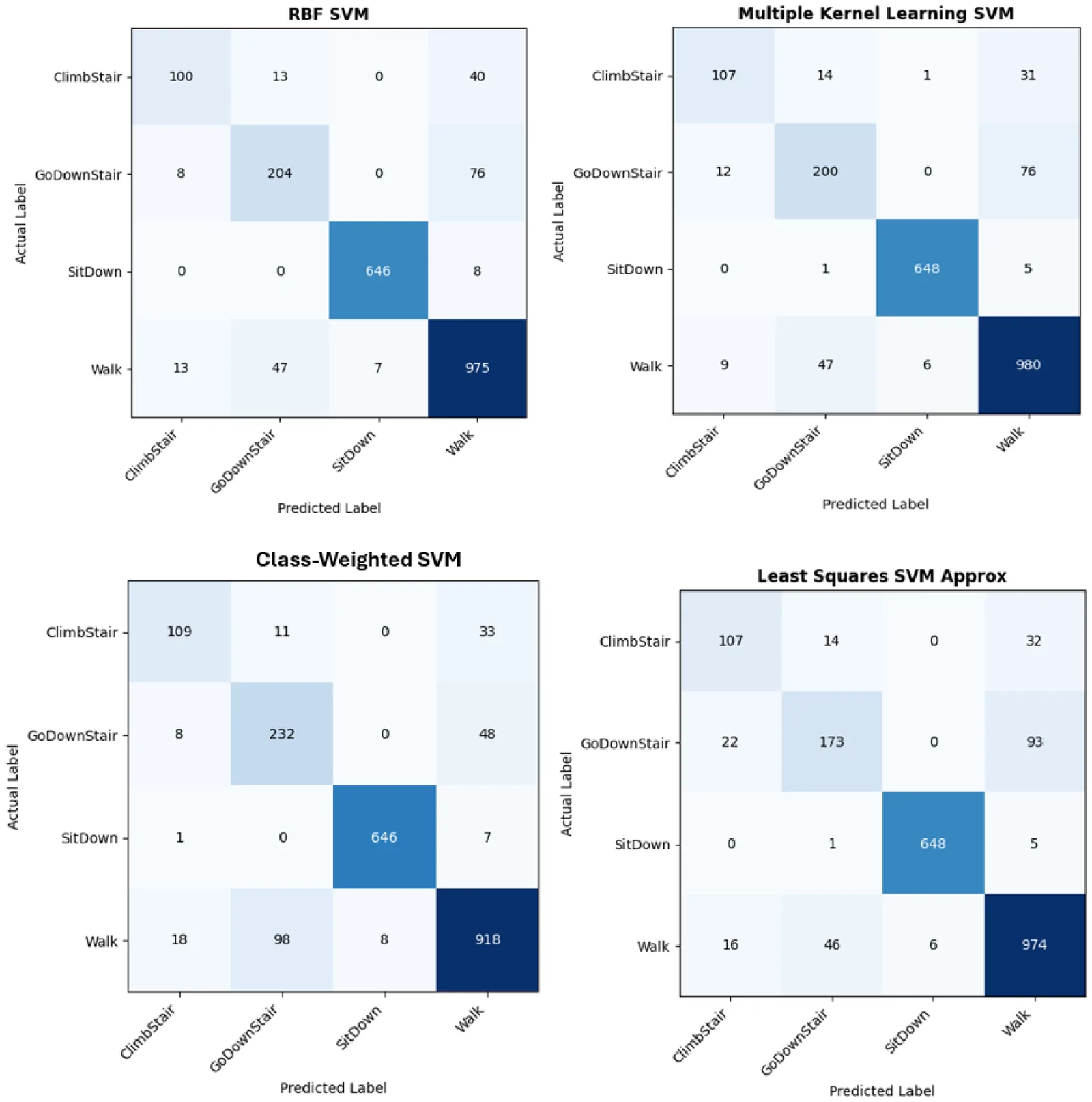
Confusion matrix of 4 best SVMs in test data.

As shown in Figure 4 and Table 3, the confusion matrices of the four best-performing models—RBF SVM, Multiple Kernel Learning (MKL) SVM, Class-Weighted SVM, and the Least Squares SVM approximation—are further analyzed to examine activity-specific recognition performance. The models achieved test accuracies of 90.08%, 90.55%, 89.14%, and 89.00%, respectively. Although their overall performances are relatively close, the confusion matrices reveal important differences in recognizing individual activities.

The RBF SVM demonstrated particularly strong recognition of Sit Down and Walk, correctly identifying 646 of 654 (98.78%) and 975 of 1042 (93.57%) samples, respectively. In contrast, Climb Stairs and Go Down Stairs were more challenging, with recalls of 65.36% and 70.83%, respectively. A major source of error was confusion with Walk: 40 Climb Stair samples and 76 Go Down Stair samples were incorrectly classified as Walk. This indicates that the RBF kernel effectively represents Sit Down and Walk but has greater difficulty distinguishing some stair-related movements from walking.

The MKL SVM achieved the highest overall test accuracy of 90.55%, correctly classifying 1935 of the 2137 test samples. Compared with the RBF baseline, MKL improved the recognition of Climb Stairs, increasing the number of correct predictions from 100 to 107 and reducing Climb Stair samples incorrectly classified as Walk from 40 to 31. MKL also improved Sit Down from 646 to 648 correct predictions and Walk from 975 to 980. The corresponding recalls for Climb Stairs, Sit Down, and Walk were 69.93%, 99.08%, and 94.05%, respectively. These results suggest that combining complementary kernel representations provides additional discriminative information for several activity patterns. However, the improvement was not uniform across all activities, as the recall for Go Down Stairs was slightly lower with MKL (69.44%) than with RBF SVM (70.83%). Therefore, the advantage of MKL is primarily reflected in its improved overall balance across the activities rather than superior recognition of every activity.

The Class-Weighted SVM exhibited a different classification behavior. Although its overall accuracy was lower at 89.14%, it achieved the highest recall for both Climb Stairs and Go Down Stairs, correctly identifying 109 of 153 (71.24%) and 232 of 288 (80.56%) samples, respectively. This suggests that sample weighting can be beneficial for distinguishing the two more challenging stair-related activities. However, this improvement was accompanied by a reduction in Walk recognition. The Class-Weighted SVM correctly identified 918 of 1042 Walk samples (88.10%), while 98 Walk samples were incorrectly classified as Go Down Stairs. Thus, the improved recognition of Go Down Stairs was obtained at the expense of increased confusion between walking and descending stairs.

Least Squares SVM approximation achieved a test accuracy of 89.00%. It performed particularly well for Sit Down, correctly identifying 648 of 654 samples (99.08%), and also maintained strong Walk recognition, correctly classifying 974 of 1042 samples (93.47%). Its main weakness was Go Down Stairs, for which only 173 of 288 samples were correctly recognized, corresponding to a recall of 60.07%. Notably, 93 Go Down Stair samples were classified as Walk. This substantial confusion between descending stairs and walking contributed considerably to the lower overall performance of the LS-SVM approximation.

Overall, Sit Down was the most consistently recognized activity, with approximately 99% recall across all four models. In contrast, Climb Stairs and Go Down Stairs were considerably more difficult to distinguish, indicating greater similarity or variability in their sensor patterns. The most prominent confusion occurred between the stair-related activities and Walk, particularly between Go Down Stairs and Walk. Among the evaluated models, MKL achieved the highest overall accuracy and improved the recognition of Climb Stairs, Sit Down, and Walk relative to the RBF baseline. Class-Weighted SVM was particularly effective for the two stair-related activities but produced substantially more confusion between Go Down Stair and Walk. These results demonstrate that the choice of SVM formulation influences not only overall accuracy but also the recognition characteristics of individual human activities.

### 3.3. Subject-Independent Evaluation

A subject-independent evaluation was carried out for the four best SVM models, whereby the training model included the data of two participants, and the data of the third participant was exclusively for testing.

The results show that MKL SVM achieved the highest overall accuracy at 88.80%, outperforming the standard RBF SVM by 0.79%. This modest improvement suggests that combining linear, RBF, and polynomial kernels captures complementary characteristics of the activity data more effectively than using the RBF kernel alone. The RBF SVM also performed strongly, reaching 88.01%, which indicates that the classes are largely separable through nonlinear decision boundaries. The Class-Weighted SVM and LS-SVM approximation achieved substantially lower accuracies of 80.25% and 80.01%, respectively. Class weighting may have improved sensitivity to minority activities at the expense of overall accuracy, while the approximate kernel mapping used by LS-SVM may have lost information important for separating similar activities.

### 3.4. McNemar’s Test

To further assess the difference between the two highest-performing models, McNemar’s test was applied to the paired test predictions of the RBF and MKL SVMs. In test observations, both models correctly classified 1891 samples, while 168 samples were incorrectly classified by both models as reported in Table 4. For the discordant predictions, MKL correctly classified 44 samples that were misclassified by RBF, whereas RBF correctly classified 34 samples that were misclassified by MKL. As reported in Table 5, the resulting McNemar exact test yielded (*p* = 0.3082), indicating that the difference was not statistically significant at the 0.05 level. Therefore, although MKL achieved the highest observed test accuracy (90.55%) compared with RBF SVM (90.08%), this result should be interpreted as a modest numerical improvement rather than statistically confirmed superiority. Nevertheless, the activity-specific confusion matrices indicate differences in the classification behavior of the models, demonstrating that the alternative kernel representations influence individual activity recognition even when their overall performances are statistically comparable.

## 4. Discussion

The experimental results provide important insights into the effectiveness of different SVM-based approaches for Human Activity Recognition (HAR), particularly in relation to kernel design, model complexity, and learning strategies. The findings indicate that performance improvements are primarily driven by the ability of a model to capture complex data representations rather than by increasing the number of classifiers or modifying the optimization process alone. Among all evaluated methods, the MKL SVM achieved the highest classification accuracy, slightly outperforming the RBF SVM baseline. This result shows an effective approach by combining multiple kernels to represent heterogeneous sensor data. By integrating linear, RBF, and polynomial kernels, the MKL approach is able to simultaneously model global trends, local nonlinear patterns, and feature interactions. This richer representation is particularly beneficial in HAR, where accelerometer and gyroscope signals exhibit diverse statistical properties. The observed performance gain, although moderate, demonstrates that combining complementary kernels can enhance discriminative power beyond what is achievable with a single kernel.

The RBF SVM baseline also showed strong performance, confirming its suitability for HAR tasks. The high accuracy achieved by this model suggests that nonlinear relationships dominate the underlying data structure. This aligns with the nature of human motion, which is inherently complex and difficult to separate using simple linear boundaries. Consequently, the relatively poor performance of the Multiclass SVM further emphasizes the limitations of linear models in this domain. Despite its theoretical advantages in directly handling multiclass problems, the inability of the linear formulation to capture nonlinear patterns significantly restricts its effectiveness.

The Kernel Engineering SVM demonstrated moderate improvement over linear methods by incorporating domain knowledge through sensor-specific kernel design. However, its performance remained lower than both the RBF and MKL models. This suggests that while manually designed kernels can improve representation, they lack the flexibility of adaptive kernel combinations. In contrast, MKL dynamically balances the contribution of different kernels, allowing it to better adapt to the data distribution.

The Class-Weighted SVM and Least Squares SVM approximation produced competitive results, slightly below the RBF baseline. The Class-Weighted SVM modifies the contribution of individual training samples through a weighted learning mechanism, allowing the classifier to account for differences in sample importance during optimization. This weighting operates at the classification level and does not explicitly model temporal or spectral IMU disturbances such as sensor drift or vibration. Similarly, the LS-SVM approximation simplifies the learning process through a Least Squares-based formulation, offering potential computational advantages while maintaining competitive classification performance. However, compared with the multiple-kernel approach, these methods provide less flexibility in representing heterogeneous and complex activity patterns. This may partly explain why their overall classification accuracies remained slightly below those of the RBF and MKL-based models.

The Online Incremental SVM achieved a test accuracy of 80.67%, which was lower than the nonlinear kernel-based methods. This result should be interpreted in the context of the complete model configuration. The Online Incremental SVM implemented in this study combines a linear decision function with stochastic gradient descent and an online-compatible learning formulation, whereas the RBF and multiple-kernel approaches employ substantially more flexible nonlinear representations. Its lower classification accuracy therefore reflects the combined characteristics of this configuration rather than the incremental learning mechanism alone. Importantly, Online SVM addresses different practical requirements: it enables efficient model updating as new observations become available, which is attractive for continuous and resource-constrained wearable HAR systems. The results consequently illustrate a trade-off between predictive performance and online adaptability within the evaluated configurations. The present work compares these SVM-based approaches as complete practical alternatives; isolating the individual contribution of kernel type, optimization algorithm, and incremental learning would require a dedicated ablation study.

Another important observation is the consistency between validation and test accuracies across all methods. The small differences between these metrics suggest that the models generalize well and that the hyperparameter tuning process was effective. This also indicates that the dataset is sufficiently representative and that overfitting is not a major concern in this study. As for the subject-independent evaluation on the four best models, they still demonstrated good performance despite a decrease in accuracy, with the MKL model showing the best accuracy.

Last but not least, the results demonstrate that improving feature representation through advanced kernel design is more effective than increasing model complexity or modifying learning strategies alone. In particular, the success of the MKL approach suggests that future HAR systems can benefit from combining multiple feature spaces to better capture the complexity of human motion. At the same time, simpler models such as RBF SVM remain strong baselines due to their balance between performance and computational efficiency.

## 5. Conclusions

This study presented a comprehensive comparison of seven SVM-based approaches for HAR, including Multiclass SVM, RBF SVM, Kernel Engineering SVM, MKL SVM, Class-Weighted SVM, Least Squares SVM approximation, and Online Incremental SVM. All methods were evaluated under a unified experimental framework with consistent preprocessing, hyperparameter tuning, and performance metrics to ensure a fair and reliable comparison. The experimental results demonstrate that kernel-based methods significantly outperform linear and approximate approaches in HAR tasks. In particular, RBF SVM provided a strong baseline, confirming the importance of nonlinear modeling for sensor-based activity recognition. Among all methods, the MKL SVM achieved the highest accuracy, indicating that combining multiple kernels can enhance feature representation and improve classification performance. This suggests that leveraging complementary information from different kernel functions is an effective strategy for capturing the complexity of human motion data. In contrast, methods focusing on robustness and computational efficiency, such as Class-Weighted SVM and LS-SVM, achieved competitive but slightly lower performance, highlighting that improvements in optimization alone are insufficient without enhancing feature representation. The Multiclass SVM and Online Incremental SVM showed the lowest accuracy, emphasizing the limitations of linear decision boundaries and incremental learning strategies for complex HAR datasets.

Overall, this research indicates that feature representation is the key factor in improving SVM performance for HAR, with advanced kernel design playing a central role. The MKL approach, in particular, provides a flexible and powerful framework for integrating multiple data characteristics, making it a promising direction for future research. Future work may focus on developing automated Kernel Learning strategies, integrating deep feature extraction with SVM classifiers, and extending the proposed framework to real-time and large-scale HAR applications.

## Figures and Tables

**Table 1:** Optimal hyperparameters for each method.

Method	Best Parameters
Multiclass SVM	C = 10
RBF SVM (Baseline)	C = 10, γ = 1
Kernel Engineering SVM	C = 10, γ = 1, α = 0.7 (acc), 0.3 (gyro)
Multiple Kernel Learning SVM	C = 10, γ = 1, degree = 2, weights = (linear: 0.4, RBF: 0.4, polynomial:0.2)
Class-Weighted SVM	C = 10, γ = 1
Least Squares SVM Approx	γ = 0.1, n_components = optimal, α = 1
Online Incremental SVM	α = 0.0001, penalty = L2

**Table 2:** Performance comparison of SVM methods.

Method	Validation Accuracy (%)	Test Accuracy (%)
Multiclass SVM	78.77	79.83
RBF SVM (Baseline)	89.43	90.08
Kernel Engineering SVM	85.31	87.55
Multiple Kernel Learning SVM	89.62	90.55
Class-Weighted SVM	88.49	89.14
Least Squares SVM Approx	87.65	89.00
Online Incremental SVM	80.64	80.67

**Table 3:** Activity-specific recall evaluation.

Model	Climb Stairs (%)	Go Down Stairs (%)	Sit Down (%)	Walk (%)	Overall Accuracy (%)
RBF SVM	65.36	70.83	98.78	93.57	90.08
MKL SVM	69.93	69.44	99.08	94.05	90.55
Class-Weighted SVM	71.24	80.56	98.78	88.10	89.14
LS-SVM Approx.	69.93	60.07	99.08	93.47	89.00

**Table 4:** Accuracy of subject-independent evaluation.

Model	Overall Accuracy (%)
RBF SVM	88.01
MKL SVM	88.80
Class-Weighted SVM	80.25
LS-SVM Approx.	80.01

**Table 5:** McNemar contingency table comparing RBF SVM and MKL SVM on the test set.

RBF SVM Outcome	MKL SVM Correct	MKLSVM Incorrect
RBF Correct	1891	34
RBF Incorrect	44	168
Total	1935	202

## Data Availability

The data presented in this study are available on request from the corresponding author due to privacy.
